# Fibroblast Growth Factor 9 Inhibited Apoptosis in Random Flap via the ERK1/2–Nrf2 Pathway to Improve Tissue Survival

**DOI:** 10.3390/jcm12030809

**Published:** 2023-01-19

**Authors:** Dupiao Zhang, Mazhar Ali Raza, Jianpeng Chen, Baolong Li, Wenbin Liu, Tao Han, Hede Yan, Liangfu Jiang

**Affiliations:** 1 Department of Orthopaedics, The Second Affiliated Hospital and Yuying Children’s Hospital, Wenzhou Medical University, Wenzhou 325015, China; 2 Key Laboratory of Orthopaedics of Zhejiang Province, Wenzhou Medical University, Wenzhou 325088, China; 3 The Second School of Medicine, Wenzhou Medical University, Wenzhou 325015, China

**Keywords:** random flap, fibroblast growth factor 9, oxidative stress, apoptosis, angiogenesis

## Abstract

Background: The application of random pattern skin flaps is limited in plastic surgery reconstruction due to necrosis. Fibroblast growth factor 9 (FGF9) was reported to exert a protective effect against myocardial damage and cerebral ischemia injury, but the impact of FGF9 in random flap survival is still unclear. In this study, we used a mouse model of random flaps to verify that FGF9 can directly increase flap survival area and blood flow intensity by promoting angiogenesis. Materials and Methods: In total, 84 male C57BL/6 mice weighing between 22 and 25 g were randomly divided into three groups (n = 28 each group). After skin flap operation, one group served as a control, a treatment group received FGF9, and a treatment group received FGF9+U0126. All flap samples were incised on postoperative day 7. Results: Our results showed that flap survival was significantly increased in the FGF9 group compared with that in the control group. This protective function was restrained by U0126. The results of histopathology, laser Doppler, and fluorescent staining all showed significant increases in capillary count, collagen deposition, and angiogenesis. FGF9 also significantly increased the expression of antioxidant stress proteins SOD1, eNOS, HO-1, vascular marker proteins CD31, VE cadherin, and pericyte marker protein PDGFRβ. Western blot showed that the phosphorylation degree of ERK1/2 increased after FGF9 treatment, and the expression of Nrf2, a downstream factor, was u-regulated. Western blot and immunofluorescence results of apoptosis-related proteins cleaved caspase-3, BAX, and Bcl2 showed that FGF9 inhibited apoptosis. ERK inhibitor U01926 reduced the beneficial effects of FGF9 on skin flap survival, including promoting angiogenesis, and showing antiapoptosis and antioxidative stress activities. Conclusions: Exogenous FGF9 stimulates angiogenesis of random flap and survival of tissue. the impact of FGF9 is closely linked to the prevention of oxidative stress mediated by ERK1/2-Nrf2. In the function of FGF9 in promoting effective angiogenesis, there may be a close interaction in the FGF9–FGFR–PDGFR–ERK–VE cadherin pathway. In particular, PDGFR and VE cadherin may interact.

## 1. Introduction

Because there is no limitation on axial vessels, random flap has no limitations in terms of position and direction, so is often used in reconstructive surgery to repair skin defects due various causes such as trauma, congenital disorders, cancer, and diabetes mellitus [1,2,3]. There are many factors that affect the survival of the flap, such as the blood supply of the flap, the type of the flap, and the tension at the suture. However, ischemia is a particularly troublesome issue when the length-to-width ratio of the flap exceeds 3:1 in the face or 2:1 in the trunk and extremities, which greatly limits the clinical application and efficacy of flaps. The supply of blood in random flaps includes a microvascular network at the flap pedicle and the subdermal plexus [4]. Oxidative stress resulting from insufficient blood flow is one of the key reasons for the death of random flaps [5]. Therefore, it is of great clinical significance to reduce the necrosis area of the skin flap while inhibiting oxidative stress and promoting flap angiogenesis.

The fibroblast growth factor family has long been used in skin wound repair, especially FGF1 and FGF2, which have been widely used in clinical practice [6]. FGF9 is a vital member of the FGF family and has been used in the regeneration of blood vessels [7]. The wall of new blood vessels is thin and fragile: they need the support of pericytes. FGF9 has been shown to promote the recruitment of pericytes to new blood vessels and to grow new life-long vasoreactive blood vessels in subcutaneous implants and renal tumors in mice [8,9]. However, whether it can promote the survival of random skin flaps has not been studied.

Oxidative stress leads to an imbalance between oxides and antioxidants as a disordered metabolic process [10]. Extensive ischemia at the distal of the grafted flap leads to excessive oxide aggregation; the levels of endogenous antioxidants, including heme oxygenase-1 (HO-1), endothelial nitric oxide synthase (eNOS), and superoxide dismutase (SOD), are insufficient to sustain the need for oxidative stress in the distal region of the grafted flap, ultimately leading to apoptosis [11]. In the antioxidant system, nuclear factor red-related factor 2(Nrf2) regulates oxidative stress as a key transcription factor, and its expression is activated by its upstream regulator, extracellular regulatory protein kinase (ERK) [12]. FGF9 was reported to activate ERK phosphorylation and thus promote the expression of its downstream factors [13]. Therefore, we speculated that FGF9 can mediate the process of antioxidative stress in random flaps by activating the ERK1/2–Nrf2 pathway and the ERK1/2–Nrf2 pathway, and verified it with U0126 (U0126 is an ERK inhibitor) [14]. In this study, we established a random flap model and evaluated the impact of exogenous FGF9 in promoting angiogenesis and tissue protection in random skin flaps, and we discussed whether it can promote the survival of random skin flap by inhibiting the ERK1/2–Nrf2 pathway to stimulate apoptosis induced by excessive oxidative stress.

## 2. Materials and Methods

### 2.1. Reagents and Antibodies

Please refer to the Supplementary Materials for details.

### 2.2. Construction and Grouping of Random Skin Flap Animal Model

In total, we used 84 male C57BL/6 mice weighing between 22 and 25 g that were in good health. These mice were separated into three groups in a random manner: a group serving as a control, a treatment group receiving FGF9, and a treatment group receiving FGF9+U0126. We used 1% sodium pentobarbital (50 mg/kg, i.p.) to induce anesthesia in all three groups before the surgery. Three animals in each group (n = 28) were randomly selected for LDBF detection (n = 3), and the following experiments were arranged: HE and Masson staining (n = 3), immunofluorescence (n = 6 × 3), Western blot (n = 6), and one animal for standby (in case mice gnawed their own wounds or injured each other, affecting the integrity of the flap). We selected the mouse dorsal random flap model (ratio of the pedicle to the length of the flap: 4 × 1.5 cm^2^) in the center of the mouse dorsum [3]. Subsequently, the arteries that supplies blood to this part of the flap were completely cut, and the separated flap was immediately secured with 4-0 silk. Based on the distance from the flap pedicle, we artificially divided the flap into three regions from proximal to distal: Area I, Area II, and Area III. We euthanized the mice on postoperative day (POD) 7 and immediately harvested the flap tissue.

### 2.3. Treatment Protocols

The mice in groups FGF9 and FGF9+U0126 were treated with FGF9 intraperitoneal injection at a dosage of 100 μg/kg/d for 7 d, and the control group was treated with saline in equal volumes. Mice in the FGF9+U0126 group received U0126 at a dosage of 10 mg/kg/d 1 h before FGF9 administration via injection into the peritoneal cavity [15].

### 2.4. General Evaluation of Flap Survival

On POD 3 and 7, macroscopic alterations in the hair condition, texture, color, and flap were observed with high-quality photography. The surviving region’s color was pink and the texture was soft, with new hair growth. The necrotic region was discovered to have sclerotic, black lesions devoid of new hair growth and scabs. ImageJ was used to measure each image (ver. 6.0; Media Cybernetics, Rockville, MD, USA). Three researchers participated in the data evaluation. At the time of analysis, none of the examiners knew which treatment group the samples were from [16].

### 2.5. Laser Doppler Blood Flow (LDBF) Imaging

The flap’s blood supply and vascular flow were measured using laser Doppler imaging (Moor Instruments, Axminster, UK) [17]. Anesthetized mice were located in the prone position on the area of scanning, and the entire area of the flap was scanned with the laser Doppler imager. Blood flow photographs on POD 0, 3, and 7 were viewed through the given color-coded live body flow images. Perfusion units were used to quantify blood flow, and moorLDI Review software was utilized to measure blood flow (version 6.1; Moor Instrument, Axminster, UK).

### 2.6. H&E and Masson Staining

Each group of mice had tissue samples obtained for pathological investigation. Tissues were treated with 4% paraformaldehyde for fixation, then paraffin-embedded, and sectioned into 5 μm for Masson and H&E staining. We investigated sections under a light microscope to evaluate histological alterations. Three researchers specializing in histopathology participated in the data evaluation. At the time of analysis, none of the examiners knew which treatment group the samples were from.

### 2.7. Immunofluorescence

After dewaxing and antigen repair, the sections were incubated in pure water with 5% BSA at 37 °C for 30 min, and then with primary antibody at 4 °C for 12 h. Next, we washed the tissues 3 times. Afterward, we applied the corresponding secondary antibodies (1: 500), and light was avoided for 1 h. Finally, we washed the tissues 3 times for 5 min each. We sealed the tablet with sealing solution including DAPI. Photographs were taken with a fluorescence microscope (Nikon, Tokyo, Japan).

### 2.8. Western Blot Analysis

Tissues were isolated and rapidly stored at −80 °C. Tissues were treated with lysate containing phenylmethanesulfonyl fluoride (PMSF) and phosphatase inhibitor for 30 min. Tissue was crushed with a grinding machine. After centrifugation at 12,000 rpm for 10 min, then Coomassie bright blue method was used to determine the protein concentration. Proteins were loaded per lane onto a gel and then transferred to a PVDF membrane (Bio-Rad, Hercules, CA, USA). Primary antibodies were incubated with the membrane for 12 h at 4 °C after a 2 h preincubation with 5% milk (Bio-Rad). Next, the membranes were inserted into the incubator for 2 h with HRP-conjugated secondary antibodies after being washed three times with TBST. A ChemiDoc XRS+ Imaging System (Bio-Rad, Hercules, CA, USA) received the upregulated signals.

### 2.9. Statistical Analysis

Analysis of variance was used to conduct statistical analysis. Turkey’s post hoc analyses were utilized when an experiment was planned to compare data from more than two groups. Two-sided Student’s *t*-test was used when comparing only two groups in an experiment. SPSS (version 19.0, Chicago, IL, USA) was utilized to conduct statistical analysis. The representation of the data was mean ± standard deviation (SD). *p* < 0.05 indicates statistical difference.

## 3. Results

### 3.1. FGF Could Enhance Random Flaps’ Survival Rate

**Figure 1:**
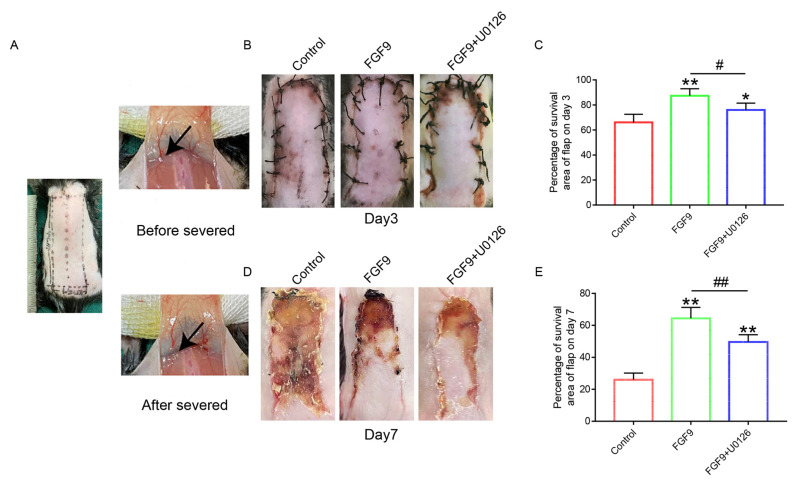
FGF9 application directly elevated the flaps’ survival area. (**A**) Photographs of flap and modeling area before and after vascular blockage. The black arrow refers to the part where the blood vessel is cut off (**B**,**D**) Photographs reporting the survival of flap at 3 and 7 days following to operation in control, FGF9, and FGF9+U0126 groups (3 days: 66.00%, 86.67%, and 76.17%; 7 days: 26.0%, 64.5%, and 49.7%, respectively). (**C**,**E**) Graph of the skin flap stock region percentage at 3 and 7 days for each group. The data are presented as the mean values ± SD, n = 3. * and ** indicate *p <* 0.05 and *p <* 0.01 against the control group, respectively; # and ## indicate *p <* 0.05 and *p <* 0.01 comparing the FGF9 group to the FGF9+U0126 group, respectively indicating statistical significance.

Necrosis occurred in Area I of the flap, and tissue was wrinkled, dry, rigid, and dark. The necrosis gradually extended to the pedicle of flap (Figure 1A). The flap survival in the FGF9 group was slightly better than that of the control group on POD 3 (Figure 1B,C). On POD 7, the flap survival area percentage in the FGF9 group remained at 60%, which was much better than that of control group (Figure 1D,E). Furthermore, compared with the FGF9+U0126 group, we found that the flap survival area in the FGF9+U0126 group was worse than that of the FGF9 group on POD 3 and 7 (Figure 1B–E). These results suggested that FGF9 could effectively protect random flaps’ survival, and this protective impact was linked to ERK and its downstream proteins.

### 3.2. FGF9 Could Promote Vascular Regeneration in Random Flaps 

**Figure 2:**
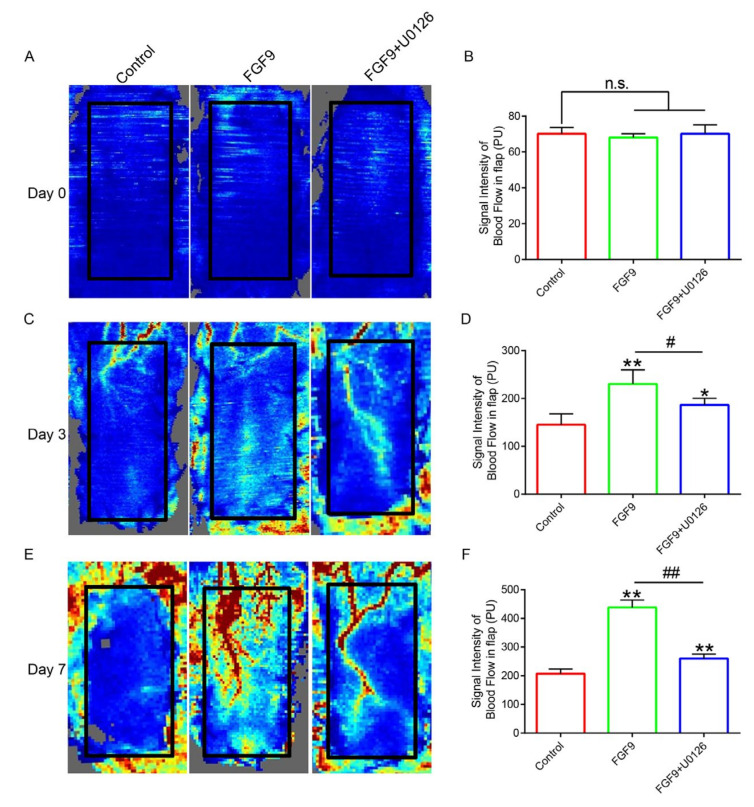
FGF9 accelerates blood flow reconstruction of flap. (**A**,**C**,**E**) The LDBF technique indicated the immediate postoperative subcutaneous blood flow, as well as three and seven days after surgery. (**B**,**D**,**F**) The statistical results of blood flow signal intensity of skin flap in the control group, FGF9 group and FGF9+U0126 group at 3 and 7 days after operation l (3 days: 142.67, 228.33, and 180; 7 days: 209.00, 456.00, and 256.67). The data are reported as the mean values ± SD, n = 3. * and ** indicate *p <* 0.05 and *p <* 0.01 against the control group, respectively; # and ## indicate *p <* 0.05 and *p <* 0.01 comparing the FGF9 group to the FGF9+U0126 group, respectively, demonstrating statistical significance. n.s. represents not statistically significant.

The key to flap survival is adequate blood supply, and we used LDBF to demonstrate microvascular network reconstruction. The results revealed that the strength of the blood flow signal in the FGF9 group was slightly stronger than that in the control and FGF9+U0126 groups on POD 3 (Figure 2C,D). However, on POD 7, the signal intensity of the blood flow in the FGF9 group increased to more than 400 PU, which was apparently stronger than that of the control and FGF9+U0126 groups (Figure 2E,F). These results revealed that FGF9 can efficiently induce the recovery of blood supply in random flaps.

### 3.3. FGF9 Could Promote Functional Angiogenesis 

**Figure 3:**
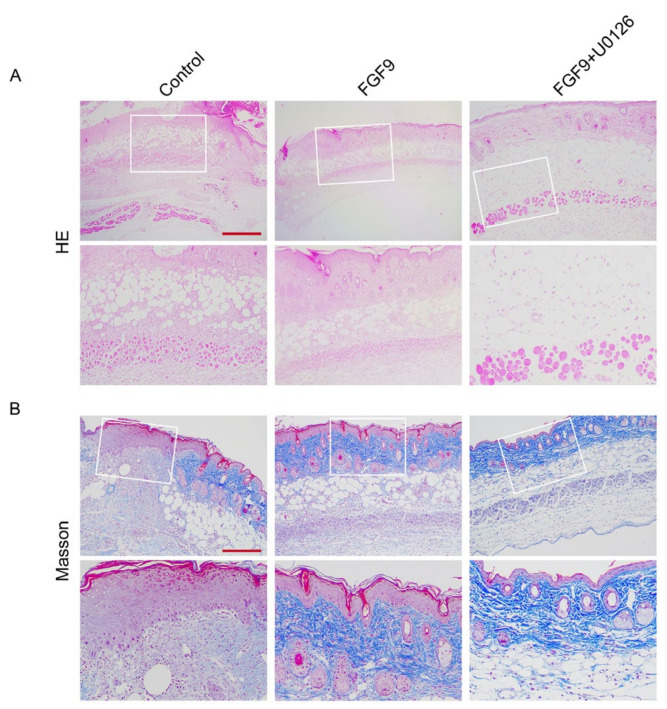
Treatment with FGF9 led to better flap histological condition. (**A**) HE staining photographs of control, FGF9, and FGF9+U0126 groups. Magnification = 4×; scale bar = 500 μm. The white-bordered region was captured at high magnification as the illustrative photograph; scale bar = 200 μm. (**B**) Blood vessels and collagen fibers were visualized by Masson trichrome stain photographs of each group. Scale bar = 500 μm. High-power photographs were also captured; scale bar = 200 μm.

**Figure 4:**
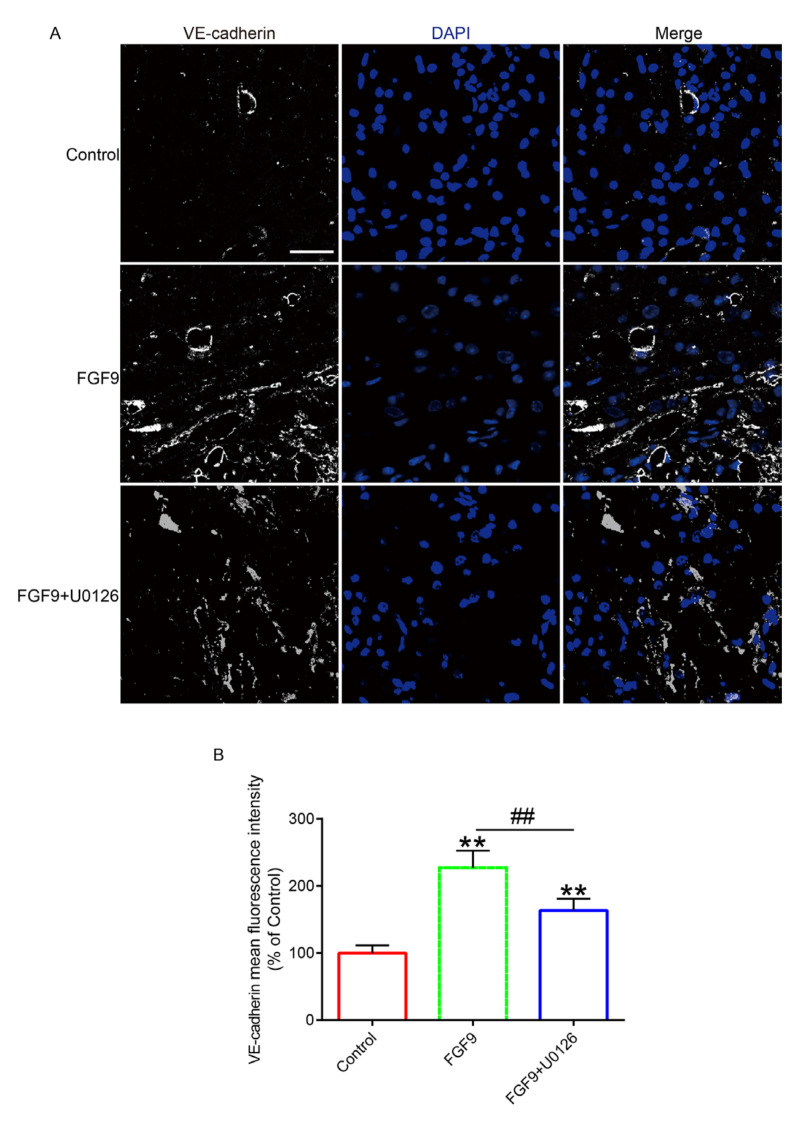
FGF9 promoted flap angiogenesis. (**A**) Immunofluorescence staining of VE cadherin protein in control, FGF9 treatment, and FGF9+U0126 treatment groups. DAPI (blue) was used to labeled nuclei. Magnification = 80×; scale bar = 25 μm. (**B**) VE cadherin fluorescence intensity statistics. ** represents *p <* 0.01 versus the control group, and ## represents *p <* 0.01 comparing the FGF9 group with the FGF9+U0126 group, indicating statistical significance. Data are the mean values ± SD, n = 6.

**Figure 5:**
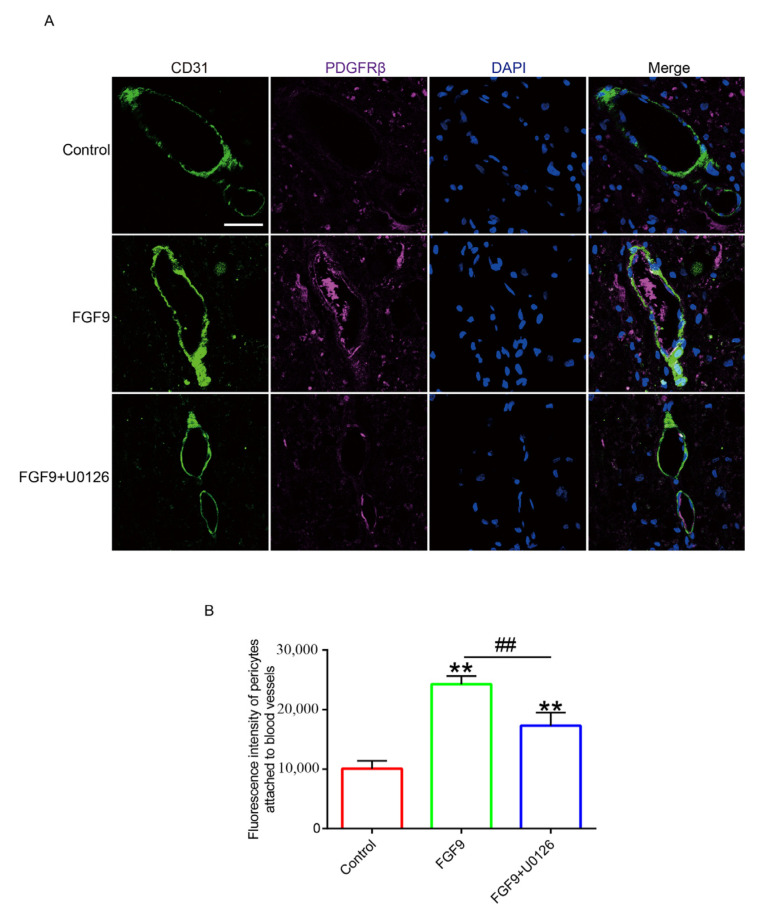
FGF9 improved vascular stability in random flaps. (**A**) Immunofluorescence staining of CD31 (green) and PDGFRβ (purple) in each group. DAPI(blue) was used to label nuclei. Magnification = 80×; scale bar = 25 μm. (**B**) CD31 and PDGFRβ fluorescence intensity statistics. ** represents *p <* 0.01 versus the control group, and ## represents *p <* 0.01 comparing the FGF9 group with the FGF9+U0126 group, indicating statistical significance. Data are the mean values ± SD, n = 6.

The key to enhancing blood perfusion is angiogenesis. Using histological staining, random flap revascularization was evaluated. H&E staining revealed that there was no evident angiogenesis in the control group’s flaps. On POD 7, there were many microvessels in the FGF9 group. FGF9+U0126 group had fewer microvessels in the skin flaps than the FGF9 group (Figure 3A). Masson trichromatic staining showed vascular infiltration and compact and orderly arrangement of collagen fibers in FGF9 group; we observed irregular and loose arrangements of collagen fibers in the control and FGF9+U0126 groups (Figure 3B). Then, we found that, as protein-promoting angiogenesis, VE cadherin levels in the FGF9 group were apparently greater than in the control and FGF9+U0126 groups (Figure 4). Furthermore, blood vessels were labeled with CD31, and pericytes were labeled with PDGFRβ. We found that there was no pericyte attachment around the vascular endothelial cells in the control group, while there was a large amount of pericyte attachment around the vascular endothelial cells in the FGF9 group. In addition, the FGF9+U0126 group had fewer pericytes attached around the vascular endothelial cells than FGF9 group (Figure 5). These results proved that FGF9 could effectively promote angiogenesis and promote pericytes to wrap vessels and maintain the stability of vessels. This function of FGF9 is closely related to the activation of ERK. 

### 3.4. FGF9 Inhibited Oxidative Stress by Mediating ERK1/2–Nrf2 Pathway 

**Figure 6:**
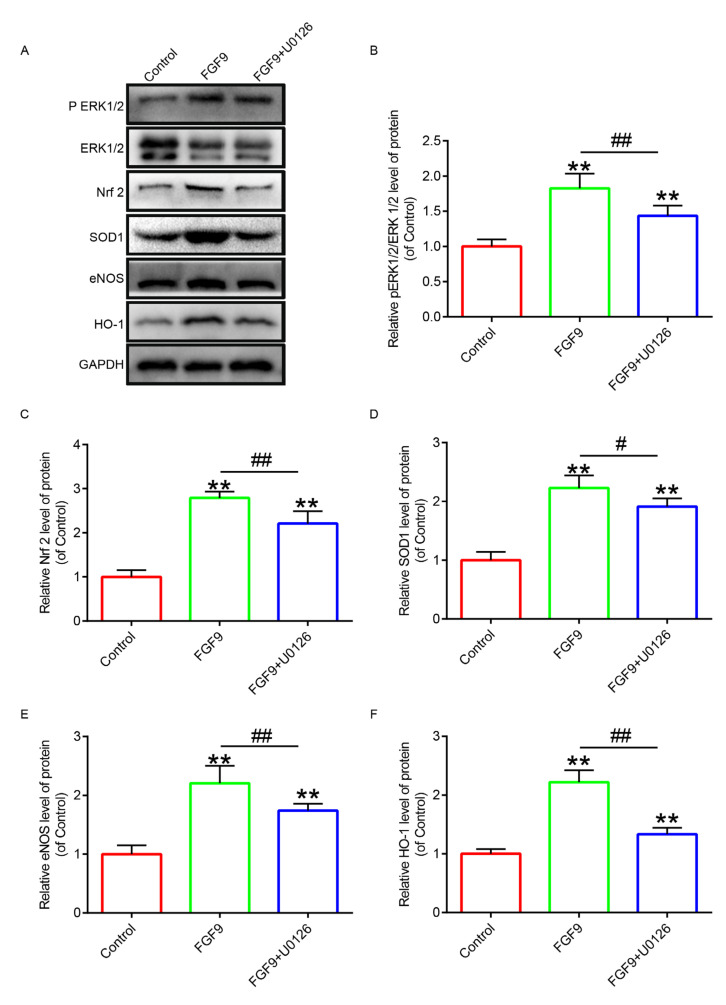
FGF9 significantly inhibited oxidative stress in random flaps. (**A**) Western blotting images of ERK1/2, Enos, HO-1, and SOD1 proteins. (**B**–**F**) Quantitative statistics of the optical density values of the these proteins. ** represents *p <* 0.01 versus the control group; # and ## represent *p <* 0.05 and *p <* 0.01 comparing the FGF9 group with the FGF9+U0126 group, respectively, indicating statistical significance. Data are the mean values ± SD, n = 6.

Oxidative stress is closely related to blood supply. In order to explore random flaps’ oxidative stress, we used Western blotting to detect the relevant indicators (Figure 6A). The results revealed that compared with the control group, the phosphorylation degree of ERK1/2 was increased with FGF9 treatment, and the expression of downstream factor Nrf2 was upregulated to enhance the expressions of eNOS, HO-1, and SOD1. In the FGF9+U0126 group, due to U0126 inhibiting the phosphorylation of ERK1/2, we found that the expression of was Nrf2 reduced compared with that of the FGF9 group, resulting in decreased synthesis of eNOS, HO-1, and SOD1 (Figure 6B–F). These results proved that FGF9 promoted the phosphorylation of ERK1/2 through the activation of the ERK1/2–Nrf2 pathway to inhibit oxidative stress. 

### 3.5. FGF9 Inhibited the Development of Apoptosis by Inhibiting Oxidative Stress

**Figure 7:**
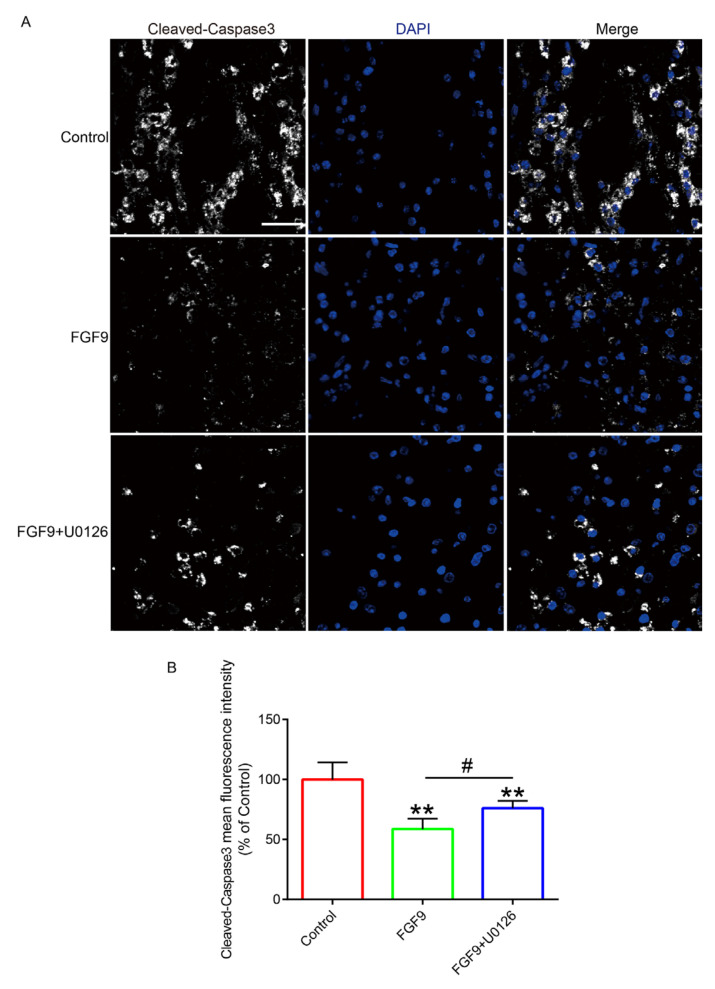
The application of FGF9 effectively reduced the number of cleaved caspase 3-positive cells in tissues. (**A**) Cleaved caspase-3 staining (white) of each group; the nuclei were labeled by DAPI (blue). Magnification = 80×; scale bar = 25 μm. (**B**) Cleaved caspase-3 fluorescence intensity statistics. ** represents *p <* 0.01 versus the control group; # represents *p <* 0.05 comparing the FGF9 group to the FGF9+U0126 group, showing statistical significance. Data are the mean values ± SD, n = 6.

**Figure 8:**
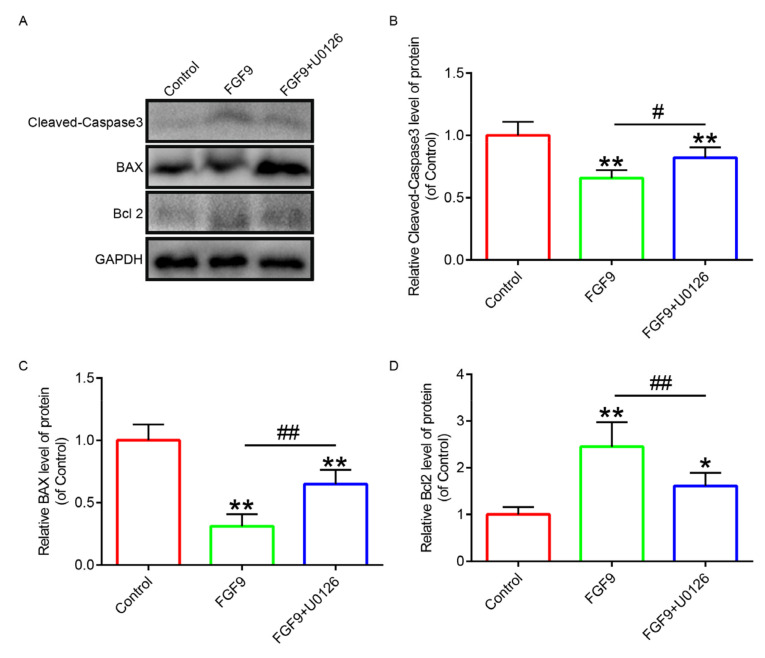
FGF9 promoted the upregulation of cleaved caspase-3, Bax, and Bcl2 expressions. (**A**) Western blotting images of the above three apoptosis-related proteins. (**B**–**D**) Quantitative statistics of the optical density values of the three proteins. * and ** indicate *p <* 0.05 and *p <* 0.01 against the control group; # and ## represent *p <* 0.05 and *p <* 0.01, respectively, comparing the FGF9 group with the FGF9+U0126 group, indicating statistical significance. Data are the mean values ± SD, n = 6.

In order to investigate the survival status of the flaps after FGF9 inhibited oxidative stress, we labelled the apoptotic cells with cleaved caspase-3 (C-C3) fluorescence staining. Immunofluorescence results displayed that the FGF9 group had the fewest C-C3-positive cells, which was apparently lower than that in the other two groups (Figure 7). In order to further verify the effect of FGF9 on apoptosis, we detected C-C3, Bax, Bcl2, and other indicators by Western blotting (Figure 8A). The experimental results suggested that the expressions of C-C3 and Bax were high in the control group, the expressions of C-C3 and Bax were downregulated in the FGF9 group, the expressions of C-C3 and Bax in the FGF9+U0126 group were upregulated compared with those in the FGF9 group, and the expression of Bcl2 was completely opposite to the trend in C-C3 and Bax (Figure 8B–D). These results indicated that FGF9 can efficiently inhibit the apoptosis induced by oxidative stress. 

## 4. Discussion

The random-pattern skin flap is a convenient tool in tissue reconstruction [1]. However, one of the most prevalent issues after random flap surgery is avascular necrosis. The main cause of ischemia and necrosis of a distal flap is blood supply insufficiency. Oxidative stress and cell apoptosis are the essential mechanisms resulting in skin flap necrosis, which lead to further tissue damage and necrosis. In our study, the results suggested that FGF9 is a core factor in random flap survival, and exogenous FGF9 enhances random flap survival by preventing oxidative stress, promoting angiogenesis and subsequently inhibiting apoptosis.

FGF9 is a core factor in biological development and tissue repair [7]. In our experiment, we found that FGF9 could significantly enhance random flap survival, and the rate and degree of tissue necrosis were significantly decreased compared with those of the control group. Interestingly, we also found that the flap-protective function of FGF9 was inhibited to a certain extent after the use of ERK inhibitor U0126. This suggested that FGF9 can protect the skin flap by activating ERK and regulating its downstream factors.

ERK, a serine threonine kinase, is linked to the control of cell differentiation, proliferation, and cytoskeleton dynamics [18]. ERK is the downstream target of FGF receptor (FGFR), and FGF activates ERK to promote its phosphorylation after acting on FGFR [19]. Oxidative stress is regulated by multiple intracellular signaling cascades, including the ERK1/2–Nrf2, JAK–STAT, and PI3K–Akt pathways [20]. Among them, the ERK1/2–Nrf2 signaling is considered to be the pivotal molecular regulatory mechanism against oxidative-stress-induced injury [21]. Nrf2 is a key antioxidant defense factor that works with antioxidant response element (ARE) to maintain normal oxidation levels [22]. A growing body of evidence suggests that ERK1/2 activates the downstream transcription factors of Nrf2 and promotes bone and nerve repair [23]. However, there was no clear evidence to confirm whether the antioxidant enhancement by FGF9 drugs in randomized flaps was strongly associated with the ERK1/2–Nrf2 signaling pathway. In the current study, we found that FGF9 significantly upregulated ERK phosphorylation and activated the Nrf2 signaling pathway compared with those of the control group. In addition, U0126 significantly reversed the impact of FGF9 on ERK1/2 phosphorylation and Nrf2 activation. In conclusion, these results suggested that the enhanced antioxidant capacity of FGF9 might prevent the flap necrosis through stimulation of the ERK1/2–Nrf 2 pathway.

Sufficient blood perfusion is a main factor promoting flap survival, and VE cadherin is a key factor promoting angiogenesis and maturation [4]. In our study, we found that FGF9 could significantly upregulate the expression of VE cadherin in random flaps, effectively promoting angiogenesis. At present, evidence increasingly showing that the survival time of new blood vessels is short and the attachment of pericytes is needed to promote the maturation of new blood vessels [16]. In our study, we found a large number of perivascular pericytes in the FGF9 group and almost no pericytes in the control group. Interestingly, compared with ERK inhibitor therapy, the number of blood vessels and pericytes in FGF9+U0126 group decreased, and VE cadherin expression was also downregulated. It was reported that FGF binds to FGFR to stimulate pericyte proliferation and to coordinate PDGFRβ signal transduction and vascular recruitment [24]. As an upstream indicator of ERK, the activation of PDGFR may promote the activation of ERK, while VE cadherin, as an adhesion factor, was reported to be regulated by ERK [25]. Therefore, we speculate that FGF9 acts on FGFR to activate its downstream PDGFR, and PDGFR further promotes the phosphorylation of ERK. VE cadherin expression was upregulated with the increase in ERK phosphorylation. However, ERK is downstream of PDGFR, and the number of PDGFRβ-positive pericytes attached to blood vessels decreased after the use of ERK inhibitor U0126. In our previous study, we found that adhesion between pericytes and blood vessels was closely related to the expression of cadherin, so we speculate that the inhibition of ERK phosphorylation with U0126 leads to the downregulation of VE cadherin, which may lead to a reduction in the number of pericytes adhering to blood vessels [26].

The continuous progression of apoptosis is the main reason for the necrosis of random flaps. Therefore, the ultimate goal of drug therapy is to inhibit the progression of apoptosis in random skin flaps [27]. In our study, we confirmed that FGF9 can effectively inhibit the progression of apoptosis after inhibiting oxidative stress. 

## 5. Conclusions

To summarize, we proved that exogenous FGF9 stimulates the angiogenesis of random flaps and the survival of tissue. FGF-family-related recombinant proteins are widely applied in clinical treatment, showing the practical significance of our results. Our study confirmed that the defensive impact of FGF9 is closely linked to the prevention of oxidative stress mediated by ERK1/2–Nrf2. We also discussed the role of FGF9 in promoting effective angiogenesis, and there may be a close interaction in the FGF9–FGFR–PDGFR–ERK–VE cadherin pathway. In particular, PDGFR and VE cadherin may interact. However, the specific mechanism still requires further study. 

## Data Availability

The data used to support the findings of this study are available from the corresponding author upon request.

## Supplementary Materials

The following supporting information can be downloaded at: https://www.mdpi.com/article/10.3390/jcm12030809/s1, Supplementary Materials S1: Reagents and antibodies.
