# Supplementary material for: Fibroblast Growth Factor 9 Inhibited Apoptosis in Random Flap via the ERK1/2–Nrf2 Pathway to Improve Tissue Survival
Source: J Clin Med. 2023 Jan 19;12(3):809. doi: 10.3390/jcm12030809 (PMC9917905; doi:10.3390/jcm12030809)
Supplement: Supplementary file 1 [file jcm-12-00809-s001.zip › jcm-2110749-supplementary.pdf]

Supplementary Material：Antibody reagents in the experiment

1. FGF9 was supplied by PeproTech (NJ, USA).
2. U0126 was supplied by MCE (NJ, USA).
3. Primary antibodies to PDGFR β, CD31, Nrf2, SOD1, eNOS and HO-1 were supplied by Abcam (Cambridge, UK).
4. Primary antibodies to Cleaved-Caspase 3, pERK1/2, Bax and Bcl2 were supplied by zenbio (Chengdu, China).
5. Primary antibodie to VE cadherin and ERK1/2 were obtained from Affinity Biosciences (OH, USA).
6. Anti-rabbit and anti-mouse secondary antibodies IgG-conjugated with horseradish peroxidase (HRP), or with Alexa Fluor647 (infrared) and Alexa Fluor488 (green) were from Abcam (Cambridge, UK).
7. An enhanced chemiluminescence (ECL) kit was provided by Bio-Rad (Hercules, CA, USA).
8. DAPI (4′,6-diamidino-2-phenylindole), a fluorescent agent for cell nuclear staining was from Sigma-Aldrich (St. Louis, MO, USA).
